# Effect of Ultraviolet Light C (UV-C) Radiation Generated by Semiconductor Light Sources on Human *Beta*-Coronaviruses’ Inactivation

**DOI:** 10.3390/ma15062302

**Published:** 2022-03-20

**Authors:** Piotr Sobotka, Maciej Przychodzki, Konrad Uściło, Tomasz R. Woliński, Monika Staniszewska

**Affiliations:** 1 Faculty of Physics, Warsaw University of Technology, 00-662 Warsaw, Poland; Maciej.przychodzki.stud@pw.edu.pl (M.P.); tomasz.wolinski@pw.edu.pl (T.R.W.); 2 Centre for Advanced Materials and Technologies CEZAMAT, Warsaw University of Technology, 02-822 Warsaw, Poland; konrad.uscilo.stud@pw.edu.pl

**Keywords:** human coronavirus OC43 (HCoV-OC43), severe acute respiratory syndrome coronavirus 2 (SARS-CoV-2), coronavirus disease 2019 (COVID-19), ultraviolet C (UV-C), light-emitting diode (LED), virus inactivation

## Abstract

The severe acute respiratory syndrome coronavirus 2 (SARS-CoV-2) pandemic has completely disrupted people’s lives. All over the world, many restrictions and precautions have been introduced to reduce the spread of coronavirus disease 2019 (COVID-19). Ultraviolet C (UV-C) radiation is widely used to disinfect rooms, surfaces, and medical tools; however, this paper presents novel results obtained for modern UV-C light-emitting diodes (LEDs), examining their effect on inhibiting the multiplication of viruses. The main goal of the work was to investigate how to most effectively use UV-C LEDs to inactivate viruses. We showed that UV-C radiation operating at a 275 nm wavelength is optimal for germicidal effectiveness in a time exposure (25–48 s) study: >3 log-reduction with the Kärber method and >6 log-reduction with UV spectrophotometry were noted. We used real-time quantitative reverse transcription polymerase chain reaction (RT-qPCR) to reliably estimate virus infectivity reduction after 275 nm UV-C disinfection. The relative quantification (RQ) of infectious particles detected after 40–48 s distinctly decreased. The irradiated viral RNAs were underexpressed compared to the untreated control virial amplicon (estimated as RQ = 1). In conclusion, this work provides the first experimental data on 275 nm UV-C in the inactivation of human coronavirus OC43 (HoV-OC43), showing the most potent germicidal effect without hazardous effect.

## 1. Introduction

One of the methods to limit the spreading of viruses is to reduce the possibility of their multiplication on usable areas. The most frequently used method of disinfecting surfaces is the use of alcohol-based agents, but this is not the optimal solution in every situation [1]. Moreover, often the long-term use of these agents may cause discomfort or damage to the surface.

In addition to chemicals, methods such as ozonation and disinfection with UV-C radiation generated from gas-discharge lamps are also used. Unfortunately, there is no professional research available in the literature defining what parameters the UV-C radiation should have in order to achieve effective virus inactivation.

There are a few publications that analyze the effect of LED-driven UV-C radiation on inactivation of the SARS-CoV-2 virus. Papers that describe the interaction of UV-C radiation usually show high-power results obtained from a lamp where the source of the UV-C radiation is a gas discharge lamp. There are no comparative analyses for different radiation powers or wavelengths of light. The one work that touched upon the topic of using UV-C LEDs for virus inactivation [2] focused only on one wavelength and no other methods of detecting the viral load apart from the cytopathic effect were analyzed. Publications that have appeared in recent years and dealt with the topic of the use of UV-C LEDs mainly concern their use in destroying bacteria [3], fungi [3], and microorganisms [4]; in pasteurization [5]; and for the inactivation of viruses [6] (without focusing on beta-coronaviruses). The results presented in this paper are a comprehensive analysis of the possibilities of currently available UV-C LEDs for virus inactivation, their effectiveness, and optimal use.

The ultraviolet radiation wavelength range of the UV-C type is 200 nm to 280 nm [7]. For many years, this was the range reserved for discharge lamps [7,8,9]. However, modern semiconductor manufacturing techniques have recently allowed us to create alternative UV-C radiation sources to discharge lamps in the form of LEDs [10]. At the beginning stage of research regarding the influence of these UV-C LEDs on the multiplication of viruses, LEDs distributed by THORLABS with an optical power of 1 mW and a wavelength of 250 nm were available. For comparative purposes, an LED with a wavelength of 275 nm, but a different power, i.e., 1.4 W, was also used. More detailed information on the use of these light sources will be presented later.

The COVID-19 pandemic that began in early 2020 has led to increased interest in solving problems of viral transmission. In response to this situation, a number of studies have been prepared (including this one) regarding the possibility of using UV-C radiation to inactivate viruses [11,12,13]. The results presented in this paper allow us to eliminate the limitations of publications from the past. The results are comprehensive, which means that there are not only studies on light sources, but also a thorough microbiological analysis of viruses affected by light. In the case of virus multiplication analyses, efforts were made to eliminate errors and radiation losses resulting from interaction with the containers where the biological material was kept, as well as to optimize the shape of the illuminators and the sample’s exposure time to radiation. In the present study, we focused on the inactivation of ssRNA human coronavirus OC43 (HoV-OC43) upon exposure to radiation of UV-C (250–275 nm) [14] in dispersed viral suspensions in a medium. Viral inactivation was monitored using the infectivity assays in the VeroE6 cell culture (cytopathic effect and UV RNA assessment) as well as by real-time quantitative reverse transcription polymerase chain reaction (RT-qPCR) assays.

## 2. Materials and Methods

For this research, five LED illuminators were the light sources. Table 1 presents their most important parameters.

**Figure 1:**
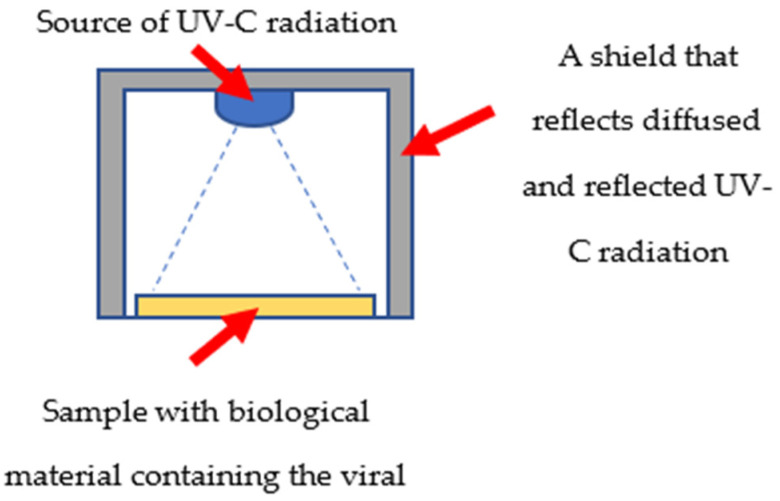
A schematic graphic diagram showing the illumination process and the signal that falls on the virus pan.

All parameters listed in Table 1 were measured before the start of microbiological measurements. LEDs 2–5 were equipped with ball lenses, which collimated the luminous flux and allowed for a beam angle of 15°. LED 1 had no optical system, and its effective light beam was at a level of 120° (the angular characteristics were not measured; this information comes from the manufacturer’s website). The pans where the solution with the virus was placed had a diameter of 3.5 cm, hence the illuminators were designed in such a way that the entire optical power was directed at the area where the virus was located (Figure 1). The illuminators are designed in the form of pipes—their outer diameter allows them to be placed on the pan, while the inner surface of the pipes allows waves to be reflected off. A LED diode was mounted in the upper part with a power supply. It was important that the height of the illuminators be selected in such a way that the surface of the illuminated area coincided as much as possible with the surface of the pans. Obviously, due to the different optics of the discussed light sources, the illuminator in diode 1 was much lower than the illuminators in LEDs 2–5. The length of the illuminators was selected in such a way that the light beam perfectly covered the surface of the sample with the virus. The illuminator with diode 1 is shorter because diode 1 did not have a focusing lens.

### 2.1. Virus Irradiation and Cytopathic Effect Evaluation

African green monkey kidney epithelial Vero E6 ATCC cells (LGC Standard, Poland infected with HCoV-OC43 ATCC (LGC Standard, Łomianki, Poland) were frozen and defrosted three times and the supernatant was centrifuged at 1500 rcf for 20 min (Eppendorf 5910E, Warsaw, Poland). Two hundred microliters of the test suspension were placed in a 3.5 cm diameter Petri dish under constant stirring during the ultraviolet C (UV-C) exposure. The viral suspensions were irradiated with radiation 9.88 (mJ/cm^2^). The time of exposure was selected so that the radiation dose was the same for each diode. Then 200 µL of the HCoV-OC43 virus particles (VP) suspension irradiated by UV LED diode was diluted 10-fold and 100 µL of suspension was inoculated onto Vero E6 ATCC (LGC Standard, Łomianki, Poland) at 5 × 10^4^ cells/mL with Eagle’s minimum essential medium (EMEM, ATCC, LGC Standard, Warsaw, Poland) seeded in the 96-well plate. Incubation was conducted at 35 °C with 5% CO_2_ (PHCBI, HeFei, Japan) for eight days to assess the cytopathic effect (CPE) using an inverted microscope (Leica DM IL LED, Wetzlar, Germany) [15]. Untreated control cells were used to validate the test. In our studies we used pH = 7.0 due to data on coronavirus-induced membrane fusion at a neutral pH [16]. The virus titer was calculated using Kärber’s formula, Equation (1):

log TCID_50_ = L − d (S − 0.5), (1)

where L = log of lowest dilution used in the test; d = the difference between log dilution steps; and S = the sum of proportion of “positive” tests (i.e., cultures showing CPE).

Viral stocks and collected samples were titrated by tissue culture infectious dose 50% (TCID_50_ mL^−1^) in the Vero E6 cells, using the Kärber formula [17]. The infectious titer reduction rates were calculated according to Equation (2):

(1 − 1/10^log10 (N0/Nt)^) × 100 (%), (2)

where Nt is the titer of the UV-C-irradiated sample and N0 is the titer of the sample without irradiation [2].

### 2.2. Determination of the HoV-OC43 Virus Particles after Irradiation Using UV Absorbance

Determination of the virus particle count was performed by UV spectrophotometry (Tecan, Männedorf, Switzerland) [18]. The HCoV-OC43 virus, irradiated or not, was suspended in the lysis buffer (1 M TRIS-EDTA, 10% SDS; pH = 7) at ratios of 1:3 (suspension of VP: lysis buffer), 1:5, 1:10, 1:50, and 1:100. The samples were incubated at 95 °C for 15 min, centrifuged briefly, and preserved on ice. We evaluated the VP in solution correlating to RNA content, quantified using a Spark microplate reader (Tecan, Männedorf, Switzerland). The UV absorbance was measured at 260 nm for HCoV-OC43 RNA content and at 280 nm for protein content. Furthermore, RNA purity was judged as 260 nm/280 nm = 2.0. The viral particle concentration was calculated using the method described by Maizel et al. [19]. The extinction coefficient was 1.1 × 10^12^ viral particles per OD 260 unit. We calculated VP using Equation (3):

VP = A260 × dilution factor × 1.1 × 10^12^/mL, (3)

where the 260 nm/280 nm ratio was 2.0 and the absorbance at 260 nm was 0.1–1.0 OD unit.

### 2.3. RT-qPCR Viral Quantification after Irradiation

Relative quantification of the HCoV-OC43 genome was performed by a one-step real-time quantitative reverse transcription polymerase chain reaction (RT–qPCR) of RNA extracted from supernatants using the Total RNA Maxi kit (A&A Biotechnology, Gdansk, Polska) [20]. The quality of total RNA was documented as the ratio A260 nm/280 nm = 2.0 using the NanoQuant Plate (Tecan, Männedorf, Switzerland) in the absorbance mode. The primer sequences (Eurofins, Siegen, Germany) used in the study were as follows: Forward: 5′-AGTATCCACCGAATGCAGTTG-3′ and Reverse 5′- GCTTCAAATGCTCAAAGGCTG-3′. Real-time one-step RT–qPCR was performed using the EXPRESS One-Step Superscript qRT–PCR Kit (Invitrogen, Carlsbad, CA, USA). The optimum annealing temperature (Ta) for the fixed primer concentration (150 mM) was achieved using the gradient in CFX96 (BioRad, Hercules, California USA). Thermal cycling was performed by BioRad CFX96 Touch Real-Time PCR (BioRad, Hercules, California USA) using low-profile eight-tube (0.2 mL) strips for PCR. RNA was stored at –80 °C until use. The RT-qPCR conditions were as follows: following the activation of the polymerase (15 min at 50 °C), there were 40 cycles of amplification (15 s at 95 °C, 30 s at 57.6 °C), and finally a melt curve was generated (65–95 °C increment 0.5 °C). Relative quantification (RQ) for each RNA sample was calculated using Equation (4):

RQ = Cq control − Cq sample,(4)

where Cq is the quantification cycle of untreated control virus and irradiated virus.

## 3. Results

### 3.1. Inactivation of Human Coronavirus HCoV-OC43 by Ultraviolet C (UVC) Exposure in Infectivity Assay

**Figure 2:**
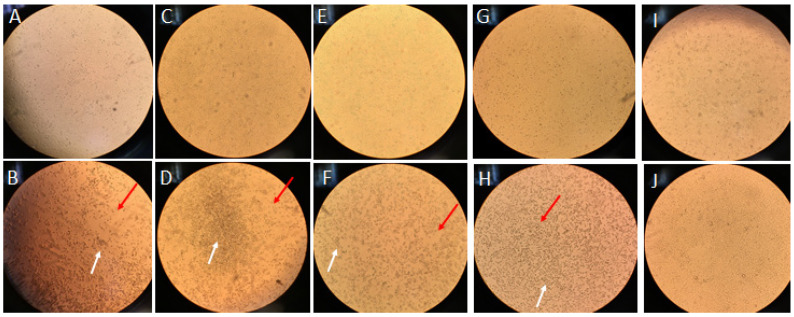
Human coronavirus (HCoV-OC43) in titer of 2.3 × 10^11^ virus particles VP/mL was irradiated with various diodes, then irradiated VP infected the VeroE6 cell cultures. A lack of cell morphological changes in the cell culture infected with irradiated HoV-OC43 was documented for diodes as follows: (**A**) J275 at 40″; (**C**) 260 at 11′40″; (**E**) 255 J at 3′45″; (**G**) 250 J at 10′17″. CPE was assessed through daily observation of infected cultures with irradiated viruses (**A**,**C**,**E**,**G**) vs. nonirradiated (**B**,**D**,**F**,**H**): pyknotic shrinking cells were noted; the white arrow points to cell rounding in a focal pattern and the red arrow points to cytoplasmic stranding. Swelling and clumping of cells was observed. Infected cells grow and clump together in “grape-like” clusters. (**I**,**J**) Uninfected cultures distinguishing normal cell changes that occur as cells age. Inverted light microscope at 100×.

Firstly, in the experimental setting, we aimed to evaluate UV-C activity, so aliquots of viral stock (200 μL, 1.6 × 10^13^ VP/mL) were placed in a plate (ø = 3.5 cm) to counteract the irradiation-derived heating of the sample for a range of times (40″, 11′40″, 3′45″, and 10′17″), corresponding to 9.88 J/cm^2^, respectively. Afterwards, 100 µL of virus suspension was back-titrated on the Vero E6 cells to determine whether the treatment eliminated all the infectious viral particles. The tissue culture infectious dose to 50% and infectious titer reduction rates were calculated and are presented in Table 2. All diodes were effective (reduction of titer ≥ 3 log) against HCoV-OC43 in the time tested (Table 2). Moreover, the cytopathic effect (CPE), referring to the structure of the VeroE6 cells caused by HCoV-OC43 infection vs. its reduction under the tested diodes, is presented in Tables S1–S4 and documented in Figure 2. In Figure 2, the HCoV-OC43-infected cell cultures, showing CPE and noninfected cells (lacking CPE), are compared with the cells infected with irradiated viruses. The reduced CPE generated by diodes was readily observed in unfixed and unstained Vero E6 cells under an inverted microscope (Leica, DM IL LED). Diodes efficiently inhibited the replication of HCoV-OC43. Moreover, the nonirradiated and irradiated virus particle concentrations using UV spectrophotometry, followed by the infectious titer reduction rates, confirmed the effectiveness of all diodes (Table 3).

### 3.2. Deactivation of Human Coronavirus HCoV-OC43 by Ultraviolet C (UV-C) 275 nm in the Time Exposure Experiment, Assessed by an Infectivity Reduction Assay

Our experiments showed that 275 nm was optimal for germicidal effectiveness in the time exposure study, as presented in Table 4. For the tested duration of UV-C 257 nm LED exposure, an effectiveness of >3 log was noted with the Kärber method and >6 log with UV spectrophotometry.

**Figure 3:**
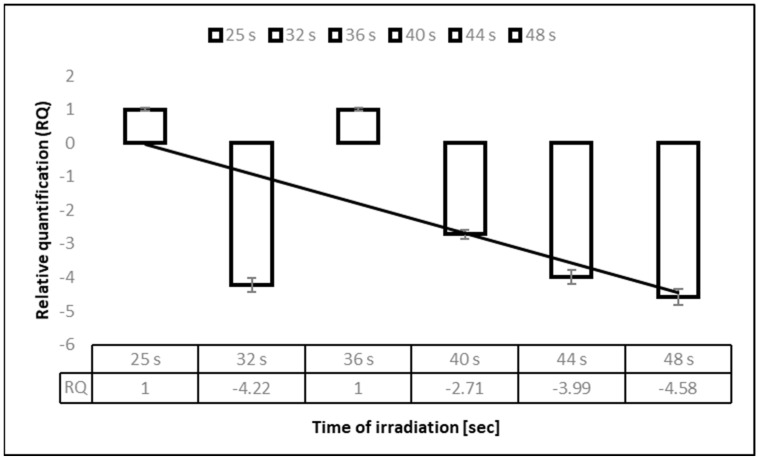
Relative quantification of viral RNA after exposure to 275 nm UV-C. Legend: RNA quality was determined by ratio of A260/ 280 = 2.0 in water free of nucleases. Quantification was performed in duplicate with the total RNA concentration being the same in every sample. Relative quantification was calculated using the formula RQ = Cq control − Cq sample, where the control was nonirradiated HCoV-OC43 harvested in a medium for seven days.

We used RT-qPCR to reliably estimate the virus infectivity reduction after 275 nm UV-C disinfection (Figure 3). It was clear that the relative quantification (RQ) of infectious particles detected after 40–48 s decreased. The values are platted in Figure 3, representing the RQ of viral RNA vs. irradiation time. The irradiated virial RNAs were underexpressed compared to the untreated control virial amplicons (estimated as RQ = 1).

## 4. Discussion

Ultraviolet radiation is key to preventing the transmission of viral pathogens on a global scale, so we tested UV-C against HCoV-OC43 (PCLII) as a representative of the beta-coronaviruses, which include SARS-CoV-2 [21]. Viral inactivation was monitored by infectivity reduction assays in the cell culture and RT-qPCR assays. The inactivation of HoV-OC43 by UV-C and the data obtained for all the parameters tested, as measured by infectivity reduction and RT-qPCR assays, are presented in Table 2, Table 3 and Table 4 and Figure 2 and Figure 3, as well as Tables S1–S5 and Figures S1–S5.

We showed that UV-C germicidal irradiation at 250–275 nm (UVGVI) [21] is effective to inactivate HCoV-OC43. A minor decay of 3 logs was achieved for HCoV-OC43 exposed to UV-C 250–275 nm radiation of 9.88 (mJ /cm^2^) in the cytopathic effect (CPE) and RNA concentration assays. The American Conference of Governmental Industrial Hygienists (ACGIH) published threshold limit values (TLVs) for 3.1 mJ/cm^2^ UV-C of 275 nm [22]. We tested UV-C 275 nm in a time exposure experiment (25–48 s). A decay of 6 log was observed for HCoV-OC43 at TLV = 6.175–11.856 mJ/cm^2^ when CPE and viral RNA concentrations were assessed (Table 2, Table 3 and Table 4 and Figure 2). Moreover, other researchers [23] showed that UV-C applied for a range of times (15, 30, and 45 min, corresponding to 1.62, 3.24, and 4.86 J/cm^2^, respectively) is sufficient to inactivate any VP in vitro on different commonly used materials.

It was noted [24] that the ssRNA genome is resistant to UV inactivation. However, in our study an inactivation of 6 log was achieved after exposure to UV-C 275 nm illumination of 9.88 J/m^2^. These inactivation kinetics are equivalent to those described in previous works [24,25,26]. Moreover, Inagaki et al. [2] considered UV-C to be the most effective viricidal region of the UV spectrum, acting through the formation of photoproducts in RNA. Thus, RT-qPCR provided an accurate quantification of viral genome copies that did not exceed the number of infective HoV-CO43 quantified simultaneously by infectivity reduction assays (CPE and UV absorbance).

## 5. Conclusions

In conclusion, this work provides the first experimental data on UV-C 275 nm in the inactivation of HCoV-OC43, showing most potent germicidal effect without any hazardous effect. Moreover, our results suggest that UV-C radiation of 9.88 J/m^2^ is sufficient to achieve 6-log inactivation of ssRNA beta-coronaviruses such as HCoV-OC43 and SARS-CoV-2. Our study demonstrated a rapid (25–48 s) inactivation rate (>3 log) for RNA viruses under UV-C irradiation.

## Figures and Tables

**Table 1:** The most important parameters of the illuminators.

Number	Manufacturer	Central Wavelength of the Light (nm)	Optical Power (μW)	$\mathrm{Optical}\;\mathrm{Power}\;\mathrm{Density}\;(\frac{mW}{cm^{2}})$	**Light Intensity Value in the Peak (lx)**	**Lens**
1	REFOND	275	7.75	0.247	1955	NO
2	THORLABS	275	0.90	0.023	49	YES
3	THORLABS	260	0.41	0.014	76	YES
4	THORLABS	255	1.40	0.044	79	YES
5	THORLABS	250	0.49	0.014	45	YES

**Table 2:** Human coronavirus (HCoV-OC43) infectious titer reduction using the UV-C diodes. Analyses based on cytopathic effect (CPE) observation.

Diode/Irradiation Time	^1^ Control (NonIrradiated) Virus	^2^ CCDI_50_	^3^ ITR
275 J/40″	10^4.0^	10^0.7^	99.9
260/11′40″		10^0.5^	99.9
255 J/3′45″		10^1.25^	99.9
250 J/10′17″		10^1.5^	99.9

Notes: CPE was recorded daily for seven days. The cell control had a complete monolayer of heathy cells. ^1^ Mean titer of nonirradiated viruses; ^2^ mean of tissue culture infectious dose 50%, calculated using the Kärber formula: log CCID_50_ = L − d (S − 0.5); where: L = log of lowest dilution used in the test; d = difference between log dilution steps; S = sum of proportion of “positive” tests (i.e., cultures showing CPE) [6]. ^3^ The infectious titer reduction rates were calculated as: (1 – 1/10 log10 ^(N0/Nt)^) × 100 (%), where Nt is the titer of the UVC-irradiated sample and N0 is the titer of the sample without irradiation [2].

**Table 3:** Infectious titer reduction of the human coronavirus (HCoV-OC43) using the UV-C diodes. Determination of the virus particle concentration by UV spectrophotometry.

Diode/ Irradiation Time	Control (NIVPC)	Irradiated Virus Particle Concentration (IVPC)	ITR
275 J/40″	2.5 × 10^13^	1.5 × 10^11^	99.9
260/11′40″		9.5 × 10^10^	99.9
255 J/3′45″		1.0 × 10^11^	99.9
250 J/10′17″		4.0 × 10^11^	99.9

Notes: Mean of particles in solution correlated to RNA content of nonirradiated virus particle concentration (NIVPC) or irradiated virus particle concentration (IVPC); UV absorbance was measured at 260 nm for its RNA content and 280 nm for its protein content. NIVPC or IVPC was calculated using the formula: VP = A260 × dilution factor × 1.1 × 10^12^/mL, where the 260/280 ratio = 1.8–2.0. The infectious titer reduction rates (ITR) were calculated as: (1 − 1/10 log10 ^(N0/Nt)^) × 100 (%), where Nt is the titer of the UV-C-irradiated sample and N0 is the titer of the sample without irradiation [2].

**Table 4:** Human coronavirus HCoV-OC43 infectious titer reduction using ultraviolet C (UV-C) 275 nm in time exposure. Analyses of cytopathic effect (CPE) and UV virial particles (VP) in infectivity assay.

Irradiation Time	^1^ Kärber’s Titer		^3^ ITR	^2^ UV Spectrophotometry		^3^ ITR
	Control NIV	CCDI_50_		Control NIVPC	IVPC	
25″	10^3.5^	10^3.3^	99.9	1 × 10^12^	1.1 × 10^11^	99.9999
32″		10^1.9^	99.9		4.6 × 10^11^	99.9999
36″		10^1.5^	99.9		4.2 × 10^11^	99.9999
40″		10^1.1^	99.9		1.9 × 10^11^	99.9999
44″		10^0.7^	99.9		5.3 × 10^10^	99.9999
48″		10^0.5^	99.9		3.7 × 10^7^	99.9999

Notes: ^1^ Recorded daily for seven days. Mean of Kärber titer of nonirradiated viruses (NIV) and mean of tissue-culture infectious dose 50% calculated using the Kärber formula: log CCID_50_ = L − d (S − 0.5), where: L = log of lowest dilution used in the test; d = difference between log dilution steps; S = sum of proportion of “positive” tests (i.e., cultures showing cytopathic effect CPE) [17]. ^2^ Mean of particles in solution correlated to RNA content of nonirradiated virus particle concentration (NIVPC) or irradiated virus particle concentration (IVPC); UV-absorbance was measured at 260 nm for its RNA content and 280 nm for its protein content. NIVPC or IVPC was calculated using the formula: VP = A260 × dilution factor x 1.1 × 10^12^/mL, where the 260/280 ratio = 1.8 − 2.0. ^3^ The infectious titer reduction rates were calculated as (1 − 1/10^log10 (N0/Nt)^) × 100 (%), where Nt is the titer of the UVC-irradiated sample and N0 is the titer of the sample without irradiation [2].

## Data Availability

All data contained within the article.

## Supplementary Materials

The following are available online at https://www.mdpi.com/article/10.3390/ma15062302/s1: Figure S1: Virus Kärber titer reduction by UV-C 275 nm in a time exposure study; Figure S2: Primer optimization at a temperature gradient of 56–66 °C; Figure S3: Products generated at melting temperature; Figure S4: Standard curve generated for the control OC43 using the primers; Figure S5: Melting peak for amplified products of OC43; Table S1: Irradiation of OC43 by diode 275 J (time of irradiation: 40″); Table S2: Irradiation of OC43 by diode ThorLabs 260 J (irradiation time: 11′46″); Table S3: Irradiation of OC43 by diode ThorLabs 255 J (irradiation time: 3′45″); Table S4: Irradiation of OC43 by diode ThorLabs 250 J (irradiation time: 10′17″); Table S5: Cytopathic effect reduction under 275 nm UV-C.
