# Supplementary material for: Effect of Ultraviolet Light C (UV-C) Radiation Generated by Semiconductor Light Sources on Human Beta-Coronaviruses’ Inactivation
Source: Materials (Basel). 2022 Mar 20;15(6):2302. doi: 10.3390/ma15062302 (PMC8949262; doi:10.3390/ma15062302)
Supplement: Supplementary file 1 [file materials-15-02302-s001.zip › materials-1604213-supplementary.pdf]

materials

MDPI

Supplementary Materials

Effect of Ultraviolet Light C (UV-C) Radiation Generated by Semiconductor Light Sources on Human Beta-Coronaviruses' Inactivation

Piotr Sobotka [1,*], Maciej Przychodzki [1], Konrad Uściło [2], Tomasz R. Woliński[1] and Monika Staniszewska [2,*]

We evaluated UVGVI effectiveness against HCoV-OC43 in the EMEM medium in plastic plates in a UV-C exposure chamber.

After irradiation we assessed the TCID value of the sample (calculated dilution) which produces CPE in fifty percent of cell cultures inoculated based on the Kärber formula. Analysis was conducted for each diodes tested, see below:

Table S1. Irradiation of OC43 by diode 275J [time of irradiation: 40″].

Virus Dilution Factor	Study 1					Study 2					Untreated Control	
10^{-1}	-	-	+	-	-	-	+	-	-	-	+	+
10^{-2}	-	-	-	-	-	-	-	-	-	-	+	+
10^{-3}	-	-	-	-	-	-	-	-	-	-	+	+
10^{-4}	-	-	-	-	-	-	-	-	-	-	+	+
10^{-5}	-	-	-	-	-	-	-	-	-	-	-	-
10^{-6}	-	-	-	-	-	-	-	-	-	-	-	-

Legend: (+) cytopatic effect; (-) lack of cytopatic effect

Study 1: Tissue-culture infectious dose to 50% ($TCID_{50}$ ml^{-1}) in the Vero E6 cells, using the Kärber formula, see below

$\log TCID_{50} = 1 – 1 * (0.2 - 0.5) = -0.7$

Virus titer: $10^{0.7}$ $TCID_{50}$/0.1 mL

Study 2: Tissue-culture infectious dose to 50% (TCID50 ml–1) in the Vero E6 cells, using the Kärber formula, see below

$\log TID_{50} = - 1 – 1 * (0.2 - 0.5) = - 0.7$

Virus titer: $10^{0.7}$ $TCID_{50}$/0.1 mL

Control - not irradiated virus

$\log TCID_{50} = - 1 - 1 * ((1 + 1 + 1 + 1) - 0,5) = - 4.5$

Virus titer: $10^{4.5}$ $TCID_{50}$/0.1 mL

Table S2. Irradiation of OC43 by diode ThorLabs 260J [irradiation time: 11′ 46′′].

**Virus Dilution Factor**	**Study 1**					**Study 2**					**Control**	
10^{-1}	-	-	+	-	-	-	-	+	-	-	+	+
10^{-2}	-	-	-	-	-	-	-	-	-	-	+	+
10^{-3}	-	-	-	-	-	-	-	-	-	-	+	+
10^{-4}	-	-	-	-	-	-	-	-	-	-	-	-
10^{-5}	-	-	-	-	-	-	-	-	-	-	-	-
10^{-6}	-	-	-	-	-	-	-	-	-	-	-	-

Legend: (+) cytopatic effect; (-) lack of cytopatic effect

Study 1: Tissue-culture infectious dose to 50% (TCID50 ml−1) in the Vero E6 cells, using the Kärber formula, see below

log $TCID_{50}$ = - 1 – 1 * (0.2 - 0.5) = - 0.7

Virus titer: $10^{0.7}$ $TCID_{50}$/0.1 mL

Study 2: Tissue-culture infectious dose to 50% ($TCID_{50}$ mL^{-1}) in the Vero E6 cells, using the Kärber formula, see below

log $TCID_{50}$ = - 1 – 1 * (0.2 - 0.5) = - 0.7

Virus titer: $10^{0.7}$ $TCID_{50}$/0.1 mL

Control - not irradiated virus

log $TCID_{50}$ = -1 -1 * ((1 + 1 + 1) - 0,5) = - 3.5

Virus titer: $10^{3.5}$ $TCID_{50}$/0.1 mL

Table S3. Irradiation of OC43 by diode ThorLabs 255J [irradiation time: 3′ 45′′].

**Virus Dilution Factor**	**Study 1**					**Study 2**					**Control**	
**10^{-1}**	+	-	+	+	-	-	+	+	+	+	+	+
**10^{-2}**	-	-	-	-	-	-	-	-	-	-	+	+
**10^{-3}**	-	-	-	-	-	+	-	-	-	-	+	+
**10^{-4}**	-	-	-	-	-	-	-	-	-	-	+	-
**10^{-5}**	-	-	-	-	-	-	-	-	-	-	-	-
**10^{-6}**	-	-	-	-	-	-	-	-	-	-	-	-

Legend: (+) cytopatic effect; (-) lack of cytopatic effect

Study 1: Tissue-culture infectious dose to 50% ($TCID_{50}$ ml^{-1}) in the Vero E6 cells, using the Kärber formula, see below

log $TCID_{50}$ = -1 -1* (0.6 - 0.5) = - 1.1

Virus titer: $10^{1.1}$ $TCID_{50}$/0.1 mL

Study 2: Tissue-culture infectious dose to 50% ($TCID_{50}$ ml^{-1}) in the Vero E6 cells, using the Kärber formula, see below

log $TCID_{50}$ = - 1 - 1* ((0.8 + 0.2) - 0.5) = - 1.5

Virus titer: $10^{1.5}$ $TCID_{50}$/0.1 mL

Control - not irradiated virus

log $TCID_{50}$ = - 1 - 1 *((1 + 1 + 1 + 0.5) - 0.5) = - 4.0

Virus titer: $10^{4.0}$ $TCID_{50}$/0.1 mL

Table S4. Irradiation of OC43 by diode ThorLabs 250J [irradiation time: 10′ 17′′].

Virus Dilution Factor	Study 1					Study 2					Control	
10^{-1}	+	+	+	+	+	+	+	+	+	+	+	+
10^{-2}	-	-	-	-	-	-	-	-	-	-	+	+
10^{-3}	-	-	-	-	-	-	-	-	-	-	+	+
10^{-4}	-	-	-	-	-	-	-	-	-	-	+	-
10^{-5}	-	-	-	-	-	-	-	-	-	-	-	-
10^{-6}	-	-	-	-	-	-	-	-	-	-	-	-

Legend: (+) cytopatic effect; (-) lack of cytopatic effect

Study 1: Tissue-culture infectious dose to 50% ($TCID_{50}$ mL^{-1}) in the Vero E6 cells, using the Kärber formula, see below

log $CCID_{50}$ = - 1 – 1 * (1 - 0.5) = - 1.5

Virus titer: $10^{1.5}$ $TCID_{50}$/0.1 mL

Study 2: Tissue-culture infectious dose to 50% ($TCID_{50}$ mL^{-1}) in the Vero E6 cells, using the Kärber formula, see below

log $CCID_{50}$ = - 1 – 1 * (1 - 0.5) = - 0.5

Virus titer: $10^{1.5}$ $TCID_{50}$/0.1 mL

Control - not irradiated virus:

log $TCID_{50}$= - 1 - 1 * ((1 + 1 + 1 + 0.5) - 0.5) = - 4.0

Virus titer: $10^{4.0}$ $TCID_{50}$/0.1 mL

Exposition time:

t_0 = 0s
t_1 = 25s
t_2 = 32s
t_3 = 36s
t_4 = 40s
t_5 = 44s
t_6 = 48s

Results of microscopic assessment of cytopathic effect of :

„+" – cytophatic effect
„-'' – lack of cytopathic effect

Table S5. Cytopathic effect reduction under UV-C 275 nm.

	Dilution -1	Dilution -2	Dilution -3
t_0	+	+	-
t_1	+	-	-
	+	+	-
	+	+	-
	+	+	-

	+	+	-
	+	+	-
	+	+	-
t_2	+	-	-
	+	-	-
	+	-	-
	+	-	-
	-	+	-
t_3	+	-	-
	+	-	-
	+	-	-
	+	-	-
	+	-	-
t_4	-	-	-
	+	-	-
	-	-	-
	+	-	-
	-	-	-
t_5	-	-	-
	-	-	-
	-	-	-
	-	-	-
	-	-	-
t_6	-	-	-
	-	-	-
	-	-	-

Kärber titer:

log $CCID_{50}$ = L – d (S – 0.5), where:

L = log of lowest dilution used in the test; d = difference between log dilution steps; S = sum of proportion of "positive" tests (i.e. cultures showing cytopathic effect CPE)

time $\boldsymbol{t_0}$:

log $CCID_{50}$ = - 1 - 1 * ((1 + 1 + 1) - 0,5) = - 3.5

virus titer: $10^{3,5}$ $CCID_{50}$ /0.1 mL

time $\boldsymbol{t_1}$:

log $CCID_{50}$ = - 1 - 1 * ((1 + 0.8)-0.5) = -3.3

virus titer: $10^{3,3}$ $CCID_{50}$ /0.1 mL

time $\boldsymbol{t_2}$:

log $CCID_{50}$ = -1 - 1 * ((1 + 0.4) - 0.5) = - 1.9

virus titer: $10^{1.9}$ $CCID_{50}$ /0.1 mL

time $\boldsymbol{t_3}$:

log $CCID_{50}$ = - 1 - 1 * ((0.8 + 0.2) – 0.5) = -1.5

virus titer: $10^{1,5}$ $CCID_{50}$ /0.1 mL

time t_4:
log $CCID_{50}$ = - 1 - 1 * ((0.6) – 0.5) = - 1.1
virus titer: $10^{1.1}$ $CCID_{50}$/0.1 mL
time t_5:
log $CCID_{50}$ = - 1 - 1 * ((0.2) – 0.5) = - 0.7
virus titer: $10^{0.7}$ $CCID_{50}$/0.1 mL
time t_6:
log $CCID_{50}$ = - 1 - 1 * (0-0.5) = - 0.5
virus titer: $10^{0.5}$ $CCID_{50}$/0.1 mL

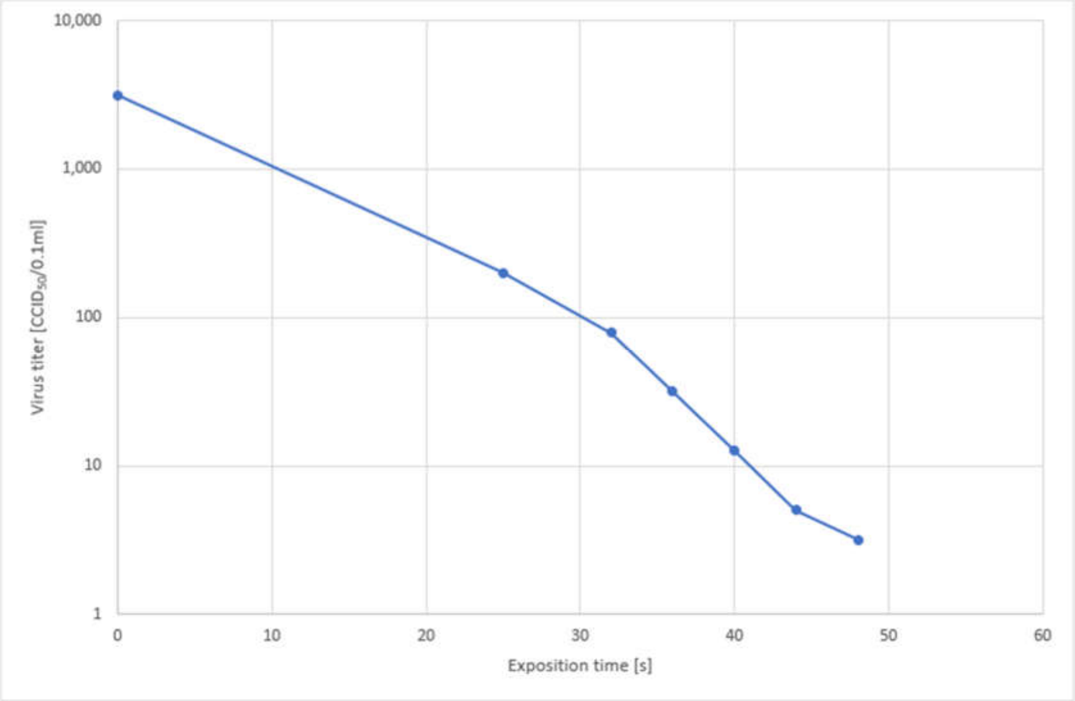

Figure S1. Virus Kärber titer reduction by UV-C 275 nm in time exposure.

The CPE decreases along with increasing exposure time. There was no unexpected viral load at t=6 in the study. A viral decline trend was observed. The best results were achieved for the time t = 6. which is in line with our predictions and the results obtained in previous studies (three repetitions). The time which can be taken into account is of the durations: t = 3, 4 and 6, since there is more than 6 log reduction of VP.

RT-qPCR analyses: quantification of virial genome after UV-C irradiation
Primers used in the study:
Human coronavirus OC43 replicase 1b gene, partial cds
1a LEFT PRIMER 5′AGTATCCACCGAATGCAGTTG 3′
1a RIGHT PRIMER 5′ GCTTCAAATGCTCAAAGGCTG 3′
PRODUCT SIZE: 150 pz

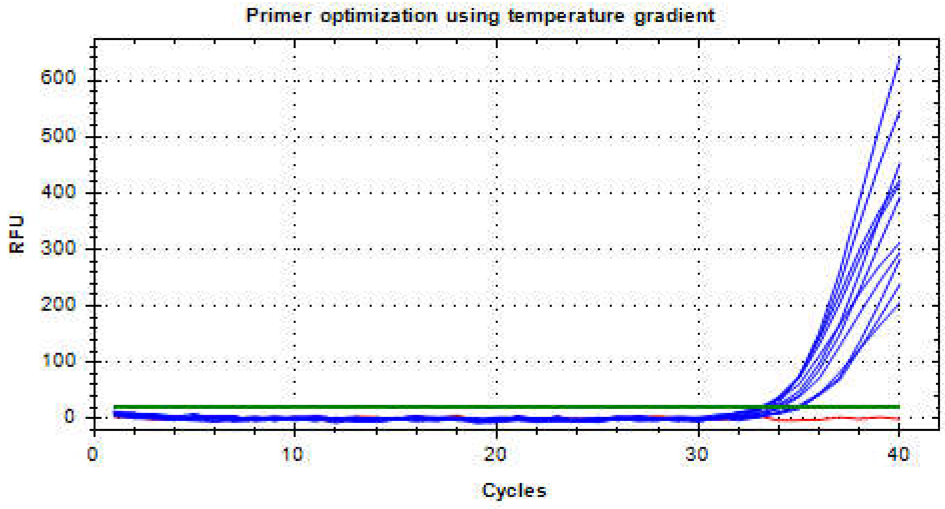

Figure S2. Primer optimization at gradient temperature starting from 56 - 66 °C. Legend: blue line represents amplification curve, red represents negative control, and green means threshold line.

We showed that the primer with melting temperature at 56.7 °C produced the best results (Fig. S3 depicts the highest peak). The GC content of the sequence gives a fair indication of the primer Tm. For all qPCR reactions controls were added.

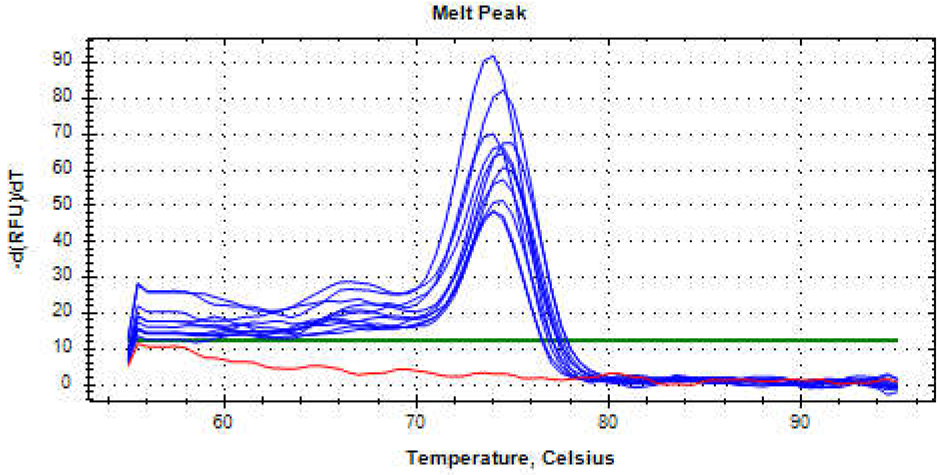

Figure S3. Products generated in melting temp. Legend: blue line represents amplification curve, red represents negative control, and green means threshold line.

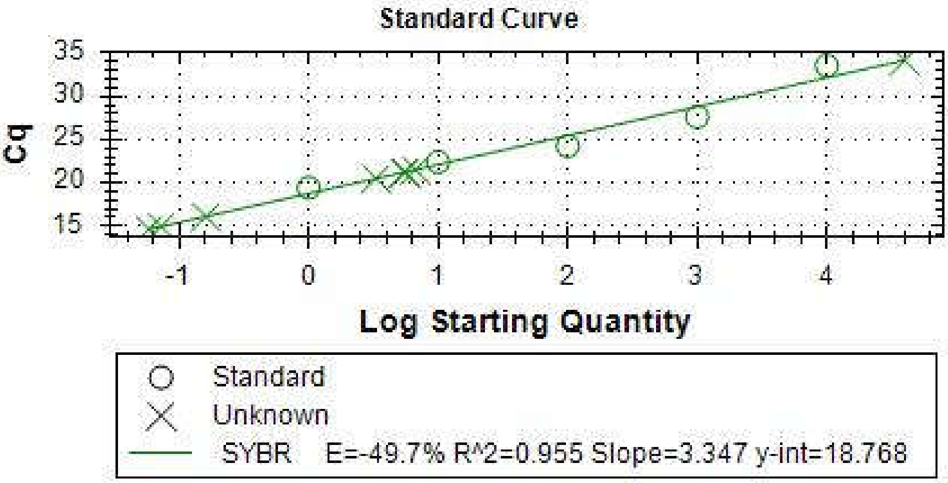

E = - 49.7% (E means efficiency)

R^2 = 0.955

Slope = 3.347

y-intercept = 18.768

Figure S4. Standard curve generated for the control HoV-OC43 using the primers.

We showed that primers amplified products with melting temperature at 78 °C (Fig. 5S).

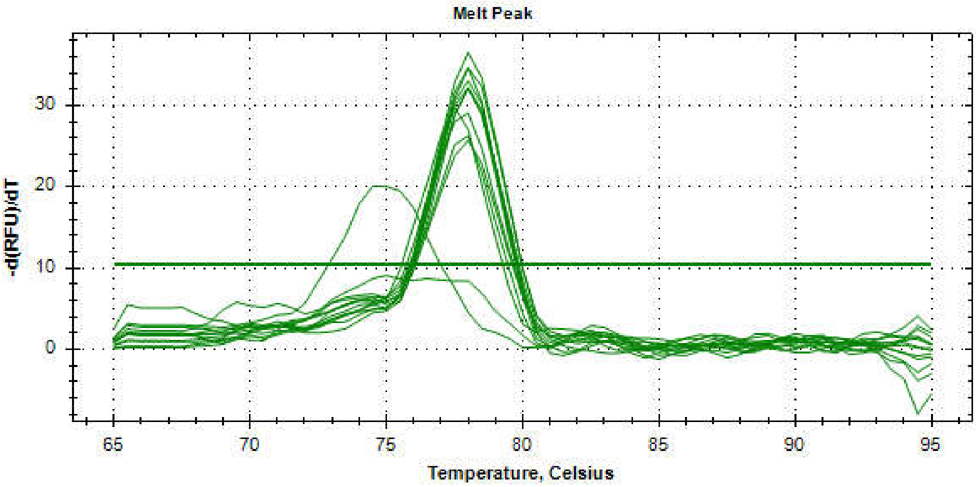

Figure S5. Melting peak for amplified products of OC43. Legend: Negative control with outstanding peak (means HoV-OC43 in medium incubated at 35 °C, 5 % CO_2 by 7 days). Green line means threshold.
